# Thyroid research: stepping forward

**DOI:** 10.1186/s13044-016-0032-z

**Published:** 2016-03-15

**Authors:** Bijay Vaidya, Andrzej Lewinski

**Affiliations:** Department of Endocrinology, Royal Devon & Exeter Hospital and University of Exeter Medical School, Exeter, UK; Department of Endocrinology and Metabolic Diseases, Medical University of Lodz and the Polish Mother’s Memorial Hospital – Research Institute, Lodz, Poland

## Abstract

It is eight years since *Thyroid Research* was launched with an aim to enhance opportunities for scientists and clinicians, working in the rapidly advancing field of thyroidology, to publish their research (Thyroid Res 1(1):1, 2008). Right from the outset, *Thyroid Research* aspired to become a prominent journal in thyroidology with high quality publications. Over the years, the journal has not only survived in the increasingly competitive field of open-access academic journal publication, it has also been making a steady progress towards achieving this ambitious goal. Now, *Thyroid Research* is ready to step forward to begin a new chapter in its publication.

So what is the change? *Thyroid Research* started as a Europe focussed journal – being initially affiliated with the Polish Thyroid Association - aiming to highlight European research and clinical advances in thyroidology across the globe [1]. Currently, the key change is to develop *Thyroid Research* into an international journal, dedicated to clinical and basic science research relating to the thyroid. With the rise of the internet in recent years, the world has become much more closely connected, and thus research carried out in any part of world could have a global impact. *Thyroid Research* will strive to become a platform for high quality research from all corners of the globe that can accessed freely by worldwide readers. To reflect this change of direction, we have initiated restructuring our Editorial Board with international representation. In addition to world-class experts, we have also taken a particular initiative to include future leaders in thyroid research in the Editorial Board. We intend to continue developing the Editorial Board in coming months.

*Thyroid Research* will continue to maintain high scientific standard by publishing only scientifically sound articles following a rigorous, fair and timely peer-review. With the rapid upsurge of open access academic journals in recent years, there is increasing condemnation that some open access journals are simply commercially driven and are prepared to publish any article for financial gain. For *Thyroid Research*, quality publication will remain a top priority. We appreciate that, for many authors, publishing their work in a journal with good impact factor is important. Therefore in the coming years, we will work towards achieving a decent impact factor for *Thyroid Research*.

We encourage you visit the newly designed website of *Thyroid Research*, and invite you to submit your high quality research in the form of original articles, short reports, reviews, commentaries and novel case-reports We also welcome your comments, criticisms and suggestions, to improve *Thyroid Research* in its new chapter and help to make it a world-class international journal.
